# An Online Tailored Self-Management Program for Patients With Rheumatoid Arthritis: A Developmental Study

**DOI:** 10.2196/resprot.4571

**Published:** 2015-12-25

**Authors:** Rixt M Zuidema, Betsie GI van Gaal, Sandra van Dulmen, Han Repping-Wuts, Lisette Schoonhoven

**Affiliations:** ^1^ Radboud University Medical Center Radboud Institute for Health Sciences Scientific Institute for Quality of Healthcare Nijmegen Netherlands; ^2^ Radboud University Medical Center Radboud Institute for Health Sciences Department of Primary and Community Care Nijmegen Netherlands; ^3^ Netherlands Institute for Health Services Research Utrecht Netherlands; ^4^ Faculty of Health Sciences Buskerud and Vestfold University College Drammen Norway; ^5^ Radboud University Medical Center Radboud Institute for Health Sciences Department of Rheumatology Nijmegen Netherlands; ^6^ University of Southampton Faculty of Health Sciences Southampton United Kingdom

**Keywords:** intervention mapping, eHealth, self-management, rheumatoid arthritis, tailoring, nursing care, early RCT

## Abstract

**Background:**

Every day rheumatoid arthritis (RA) patients make many decisions about managing their disease. An online, computer-tailored, self-management program can support this decision making, but development of such a program requires the active participation of patients.

**Objective:**

To develop an online, computer-tailored, self-management program integrated with the nursing care, as nurses have an important role in supporting self-management behavior.

**Methods:**

The intervention mapping framework was used to develop the program. Development was a multistep process: (1) needs assessment; (2) developing program and change objectives in a matrix; (3) selecting theory-based intervention methods and practical application strategies; (4) producing program components; (5) planning and adoption, implementation, and sustainability; and (6) planning for evaluation.

**Results:**

After conducting the needs assessment (step 1), nine health-related problems were identified: (1) balancing rest and activity, (2) setting boundaries, (3) asking for help and support, (4) use of medicines, (5) communicating with health professionals, (6) use of assistive devices, (7) performing physical exercises, (8) coping with worries, and (9) coping with RA. After defining performance and change objectives (step 2), we identified a number of methods which could be used to achieve them (step 3), such as provision of general information about health-related behavior, self-monitoring of behavior, persuasive communication, modeling, and self-persuasion and tailoring. We described and operationalized these methods in texts, videos, exercises, and a medication intake schedule. The resulting program (step 4) consisted of an introduction module and nine modules dealing with health-related problems. The content of these modules is tailored to the user’s self-efficacy, and patients can use the online program as often as they want, working through a module or modules at their own speed. After implementation (step 5), the program will be evaluated in a two-center pilot trial involving 200 RA patients. Log-in data and qualitative interviews will used for a process evaluation.

**Conclusions:**

The intervention mapping framework was used to guide development of an online computer-tailored self-management program via a process which could serve as a model for the development of other interventions. A pilot randomized controlled trial (RCT) will provide insight into the important outcome measures in preparation for a larger RCT. The process evaluation will provide insight into how RA patients use the program and the attrition rate.

**Trial Registration:**

Netherlands Trial Register (NTR): NTR4871; http://www.trialregister.nl/trialreg/admin/rctview.asp?TC=4871 [accessed 13-NOV-15] http://www.webcitation.org/6d1ZyIoEy

## Introduction

Rheumatoid arthritis (RA) is a chronic autoimmune disease which predominantly affects the joints. Many RA patients face physical problems such as pain, stiffness, and fatigue which cause difficulties in everyday life [1]. RA has also been linked to depression, helplessness, and anxiety and has a considerable impact on quality of life [2-5]. As life expectancy increases and the number of people living with a chronic condition increases, there has been an increase in the number of RA patients [6].

Although healthcare professionals can give patients advice and support during visits and appointments, patients have to make day-to-day decisions about management of their disease by themselves. Self-management programs can help RA patients to take an active role in the everyday management of their disease [7-9]. Self-management has been defined as the tasks undertaken by patients to manage the symptoms, treatments, lifestyle changes, and physical and psychological consequences associated with their illness [10]. Although self-management support programs are available, most programs are provided in clinical settings or in small groups [11], and not all RA patients are willing or able to participate. With a growing number of people having Internet access and the increasing use of the Internet among RA patients [12,13], an online self-management support program can be a sustainable way to support self-management behavior. Compared to face-to-face programs, online programs provide an easily accessible opportunity to reach a large group of RA patients. Also, online programs have the possibility to tailor information and can provide more anonymity than face-to-face programs. Other advantages include 24-hour availability and avoiding waiting lists [14,15].

In the Netherlands, 2 studies, one in adolescent RA patients [16] and the other focusing on work-related problems [17], have shown that the use of self-management programs is feasible for specific groups of RA patients. At this moment, there is no generic online self-management program for adult RA patients in the Netherlands. As nurses have an important role in supporting self-management behavior, such a program should preferably be integrated in the nursing care provided as part of the multidisciplinary RA care.

An online self-management program is a complex intervention. First, it should include a variety of components, such as information provision, management of symptoms, social support, and communication strategies [18]. Second, because the target population can be diverse, self-management programs should be extensive and tailored to patient needs. Within the population of RA patients there is variance in the need for self-management support, depending for example on age, level of education, gender, or work status. Third, programs should enhance patient understanding of the behavioral change required for self-management. To develop such a program requires an understanding of the factors which influence self-management behaviors.

To ensure that our development process took account of these 3 overarching requirements, we used the intervention mapping framework. We chose to develop a tailored intervention because adapting communications and behavioral change strategies to patient needs [19] means that a higher proportion of the patients receive information that is personally relevant, which increases their motivation to change their behavior [20].

This article describes the development of an online computer-tailored program and the design of an evaluation procedure using the intervention mapping framework which could serve as a guide for the development and testing of other interventions.

## Methods

The intervention mapping framework is designed to ensure that development work is focused on the most important determinants of behavior. Intervention mapping has been used successfully to develop health programs related to, for instance, medication adherence [21], promoting physical activity [22], healthy lifestyles [23], and asthma management [24]. The intervention mapping framework provides a way of systematically integrating theoretical research, empirical findings, and data collected from the population [25]. Intervention mapping provides a 6-step framework for developing health education programs: (1) identifying problem behaviors and determinants through needs assessment; (2) developing a matrix of performance objectives and change objectives; (3) selecting theory-based intervention methods and practical application strategies; (4) producing program components; (5) planning and adoption, implementation, and sustainability; and (6) planning for evaluation [25]. Active patient participation in the development process was secured by recruiting, during the first step, a multidisciplinary panel consisting of health professionals, researchers, and patients who were involved in every step of the development process.

### Step 1: Needs Assessment

First we recruited a multidisciplinary panel of 5 RA patients, 2 rheumatologists, one rheumatology nurse, a psychologist, a physiotherapist, an occupational therapist, and 3 researchers (RMZ, HRW, and BvG). The rheumatology nurse and rheumatologist played a crucial role in the development and implementation of the program.

Our needs assessment comprised two components: (1) a literature search for information on health problems, problems affecting health-related behaviors, and determinants of problems and (2) input from 2 meetings of the multidisciplinary panel. During the first meeting, we held a brainstorming session to identify the main health problems affecting RA patients. To select the most important health problems for RA patients, we coded health problems found in literature and discussed this among the multidisciplinary panel. Selection was further based on recognizability and importance of the health problems. In the second meeting, we identified problems affecting health-related behaviors and their determinants based on the literature and discussed the following questions among RA patients and health professionals: (1) why do patients have problems and (2) why do patients have problems with this behavior? In the third meeting, we asked the multidisciplinary panel whether the listed problems in health-related behavior were easily changeable or not. After these meetings, the researchers listed and coded the health problems, the problems affecting health-related behaviors, and their determinants manually.

### Step 2: Developing a Matrix of Performance Objectives and Change Objectives

In the second step, we organized the performance objectives and change objectives as a matrix to indicate which behaviors needed to change to achieve the overall goal of the program, which was to enhance patients’ ability to self-manage their disease and thus improve their quality of life. The performance objectives formalized the behavioral changes RA patients needed to make to achieve the behavioral goals of the program. The change objectives were performance objectives linked to changeable determinants of behavior. Thus, change objectives state what needs to change in determinants to achieve the performance objectives. Researcher RMZ constructed a matrix of the relationships between performance and change objectives which was subsequently validated by the multidisciplinary panel.

### Step 3: Selecting Theory-Based Intervention Methods and Practical Applications

After defining the matrix we selected theory-driven methods on the basis of behavioral change theories. In this study, 2 independent researchers linked methods from the classification of the behavior change techniques to the problems affecting health-related behaviors and their determinants in order to select methods which could be used to achieve our overall goal. The behavior change technique classification defines strategies used in supportive programs [26]. Using a summary produced by the 2 independent researchers, the multidisciplinary panel decided whether the methods were suitable for the RA patient population. We assessed the conditions under which the methods are shown to be effective to translate methods into practical applications such as texts and videos.

### Step 4: Producing Program Components

Program development was based on the change objectives and the selected theory-driven methods and consisted of composing program materials and pretesting these materials. Our research group worked with an information and communications technology partner to produce the program materials. The research group developed the content, including textual material, and our information and communications technology partner incorporated this material into an online program.

Our pretest of the online program comprised testing of the program materials by the multidisciplinary panel and testing of the program by 3 RA patients not involved in its development using the “think aloud” method [27].

### Step 5: Planning for Adoption, Implementation, and Sustainability

Intervention mapping steps 1 through 4 formed the basis of the implementation. Meetings of the multidisciplinary panel were held to identify and categorize barriers and facilitators to implementation of the online program. The rheumatologist and specialist rheumatology nurse played a crucial role in the implementation process.

### Step 6: Planning for Evaluation

In the final intervention mapping step we planned to evaluate the feasibility of the study design and the online self-management program by conducting an exploratory randomized controlled trial (RCT) and a process evaluation [28]. To do this we identified outcomes and process measures that were relevant to the program objectives. We also intend to conduct qualitative interviews with nurses, users, and nonusers of the program. Finally, we plan to monitor which topics related to the program components are discussed during nursing consultations and whether they are raised by the nurse or the patient.

## Results

### Step 1: Needs Assessment

#### Health Problems and the Underlying Behavioral Problems

We selected the 8 most important health problems in daily life for RA patients: pain, fatigue, stiffness, daily functioning, sexuality, work, social activities, and coping with RA.

We identified 9 general problems affecting health-related behavior from our literature review and through discussions among the multidisciplinary panel: (1) balancing rest and activity, (2) setting boundaries, (3) asking for help and support, (4) use of medicines, (5) communicating with health professionals, (6) use of assistive devices, (7) performing physical exercises, (8) coping with worries, and (9) coping with RA.

#### Determinants of Problem Behaviors

Our literature search determined that the following factors were relevant to problems affecting health-related behavior: knowledge, awareness, risk perception, social influence, attitude, self-efficacy, and habits. Patients confirmed the relevance of these determinants.

### Step 2: Developing a Matrix of Performance Objectives and Change Objectives

The results of the needs assessment were used to draw up a matrix of performance and change objectives. One of the performance objectives was “the patient is able to set her or his boundaries.” This performance objective was relevant to the following health problems in daily life: pain, fatigue, social activities, and work.

Next we formulated change objectives relevant to the determinants knowledge, attitude, self-efficacy, and risk perception; for example, the patient knows the consequences of not setting his or her boundaries (knowledge), and the patient is conscious of the positive consequences of setting boundaries (attitude).

### Step 3: Selecting Theory-Based Intervention Methods and Practical Applications

We used our matrix of change objectives to select a theory on which to base our intervention. The matrix placed the most emphasis on self-efficacy, attitude, and subjective norms. The theory of planned behavior posits that these constructs are the most important determinants of behavior, so we based our interventions on this theory. We also emphasize knowledge and awareness in our matrix, as these are preconditions for self-efficacy, attitude, and subjective norms. We then made a list of techniques which could be used to improve self-efficacy, attitude, subjective norms, and their preconditions.

For this, we derived the following methods per determinant from the coding manual for behavioral change techniques [26]. *Determinant knowledge*: provide general information about health behavior, increase memory and/or understanding of transferred information. *Determinant awareness*: risk-communication, self-monitoring of behavior, self-report of behavior. *Determinant social influence*: provide information about peer behavior. *Determinant attitude*: persuasive communications, belief selection, reinforcement on behavioral progress, providing contingent rewards. *Determinant self-efficacy*: modeling, practice, plan coping responses. *Determinant intention of behavior*: develop medication intake schedule. *Determinant action control*: use of social support, use of cues, self-persuasion. We operationalized these methods as follows: we used texts to increase knowledge, awareness, attitude, social influence, and action control; we used videos and exercises with feedback options to increase self-efficacy; and we encouraged patients to keep a diary within the online program to increase their awareness of their own health status and use an intake schedule to increase intention of behavior. We also tailored the program to the user’s self-reported level of self-efficacy, because self-efficacy has been found to predict changes in various health-related behaviors [29].

### Step 4: Producing Program Components

We used the change objectives and the practical applications as the basis for the online program, “Reuma zelf te lijf,” which has 10 modules consisting of 2-5 sessions each. Table 1 gives an overview of the content of the modules. The first module is the introduction module and offers a short textual introduction to the other modules as well as providing information about how the program works. After this, users can respond to a series of statements; the responses are used to tailor recommendations about which module or modules users are likely to find most helpful for improving their self-management. Examples of statements include: “I want to learn to balance my daily schedule better,” “I want to learn how to ask for support and help,” and “I want to learn how to say no to others, for example, when I'm too tired to do something.” Once users choose a module, they can work through it at their own pace, whenever they want. Every module starts with a text providing information about the topic of the module, what the patient can expect to learn from the module, and how the module is structured. Most modules allow users to respond to 2 questions to tailor the module to their self-efficacy. The responses to these questions are used to advise patients which session to move to next (session 2 for patients with a low level of self-efficacy; session 3 for patients with a high level of self-efficacy). Session 2 focuses on the following four determinants, knowledge, risk perception, awareness, and attitude, and uses informative and persuasive texts, videos of peers, and exercises to improve patients’ insight into their disease and behavior and to change their attitudes. Session 3 focuses on self-efficacy and gives users the opportunity to do exercises in familiar surroundings—for example, doing an exercise to learn how to say no to others at home with a friend. Session 4 tells users how to put the skills into practice in daily life. After each exercise, users are given the opportunity to evaluate performance by responding to a set of questions. This evaluation exercise is used to help patients identify the barriers and facilitators that are relevant to their behavior. In all exercises, it is recommended that users seeks support from their partner, family, or friends. See Multimedia Appendix 1 for an example of a module.

**Table 1:** Overview of the modules in the online program.

Modules	Number of sessions	Topics
0. Welcome	1	Short introduction to all modules and a questionnaire to assess the level of self-efficacy
1. Balancing activity and rest	4	Planning of activities
		Keeping a balance in daily life in the long term
2. Setting boundaries	5	Dare to set boundaries (say “no”)
		Setting boundaries (communicate saying “no”)
3. Asking for help and social support	4	Establishing and maintaining social contacts
		Asking for help or support
4. Use of medicines	4	How to collect information about medication
		Taking prescribed medication
5. Communication with health professionals	4	How to prepare for an appointment with a health professional
		Asking questions and/or expressing concerns during an appointment with a health professional
6. Use of assistive devices	4	Information on how to apply for assistive devices
		Deciding whether an assistive device can help you and if so, what assistive device
7. Performing physical exercises	4	Examples of physical exercises
		How to fit physical exercises into your daily life
8. Coping with worries	3	Insight into your worries
		Controlling your worries
9. Coping with RA	2	Information and tips on how to cope with RA

During the pretest the collaborative multidisciplinary panel found that the information and the exercises provided in the modules were understandable/readable and applicable. The layout and structure of the modules were described as attractive and clear. The 3 patients who tested the program using the “think aloud” method found it difficult to navigate through the program. In response to this, we adjusted the program to make navigation easier.

### Step 5: Planning for Adoption, Implementation, and Sustainability

We have planned a trial which will be conducted in 2 Dutch hospitals. The managers of the 2 rheumatology departments met regularly with the researchers to discuss trial procedures. The multidisciplinary panel identified barriers and facilitators relevant for the implementation of the online program. This information was used to design an implementation plan for the 2 hospitals which focuses on dissemination of the online program and the user's experience of interacting with the online program. We asked the specialist nurses to bring the online program to the attention of their patients during appointments. For this, a researcher explained the modules and exercises in the program to specialist RA nurses to facilitate integration of the online program with nursing care. To ensure that users’ first experiences of the program were positive, we sent potential users a written instruction manual for the program. To encourage repeated use of the program, users will be sent reminders via email.

### Step 6: Planning for Evaluation

To evaluate the feasibility, we plan to do an exploratory RCT as advised by the Medical Research Council's framework for the development and evaluation of complex interventions [30]. The aims of our feasibility study will be to evaluate the potential effectiveness of the online program for patients with RA and determine effect sizes for the various outcomes, identify outcome measures most likely to capture potential patient benefits and evaluate long-term participation and attrition rates for the online, computer-tailored self-management program [31]. Because the exploratory RCT is not expected to be powered to identify differences between groups, there is no sample size calculation. Considering the complexity of the intervention and the potentially large heterogeneity of the RA population, 200 eligible RA patients will be recruited by 2 hospitals in the eastern part of the Netherlands [NTR4871]. Inclusion criteria will be diagnosis of RA, aged 18 years or older, ability to speak and read Dutch, and access to a computer with an Internet connection. Patients receiving psychiatric or psychological treatment will be excluded. RA patients will be randomized to the intervention or control group. The control group will receive care as usual; the intervention group will have access to the online program in addition to receiving care as usual.

To explore which outcome measures are most likely to capture patient potential benefits, we chose the following potential outcomes: (1) the Patient Activation Measurement (PAM-13), which assesses the knowledge, skills, and confidence for self-management [32]; (2) the health-related quality of life survey (RAND-36), which assesses general health status in 8 dimensions (physical functioning, social functioning, role limitations [physical problems], role limitations [emotional problems], mental health, vitality, and pain [33]); (3) the Rheumatoid Arthritis Self-Efficacy scale (RASE), which measures the level of task specific self-efficacy for self-management [34]; (4) the Perceived Efficacy in Patient-Physician Interactions (PEPPI-5) [35]; (5) the short version of the self-management ability scale (SMAS-S), which measures taking initiative, investing in resources for long-term benefits, maintaining variety in resources, ensuring resource multifunctionality, self-efficacy, and maintaining a positive frame of mind [36]; (6) A scale to assess the focus on fatigue (MPCI-F) [37]; and (7) the Numeric Rating Scales (NRS), which measure pain and fatigue during the previous 2 weeks including at the moment of measurement. All instruments will be administered at baseline (T0) and after 6 (T1) and 12 months (T2). Data on the following patient characteristics will be gathered: age, gender, living situation, educational level, employment status, Disease Activity Score (DAS-28 score), physical ability using the Modified Health Assessment Questionnaire (MHAQ), time since diagnosis, current treatment, comorbidity, usage of other support programs including online programs, date of last visit to a rheumatologist, and date of last visit to a specialist nurse.

In the process evaluation we will use the framework of Saunders et al [38] to evaluate feasibility of the online program. The key components of the process evaluation are fidelity, dose received, dose delivered, reach, recruitment, and context. Data for the process evaluation will be collected from log-ins to the online program, a user questionnaire, and qualitative user interviews. The process analysis will make use of log-in data (exposure and continued use of the program), data on use of modules, and data on performance of the exercises. The user questionnaire will ask about the comprehensibility, usefulness and length of the texts and exercises, and the layout and log-in procedure. During the qualitative interviews, the frequent users and those who have stopped using the program will be asked about their reasons for using or not using the online program, which will give us insight into potential limitations and yield ideas to improve the program. We will also interview nurses to elicit their views about how introduction of the online program might affect their professional role.

Finally, to get insight into whether the online program changes the roles of patients and nurses in management of RA we have made a checklist to be completed after nursing appointments with patients in both the control group and the intervention group that covers what topics were discussed during the nursing consultation and whether it was the nurse or the patient who raised a particular topic.

## Discussion

### Strengths and Limitations

This article describes the systematically developed generic online, computer-tailored self-management supportive program for adult RA patients.

Using intervention mapping to structure the development process is a strength of this program. In the needs assessment, we successfully defined health problems, problems affecting health-related behaviors, and determinants of problems with health-related behavior which were relevant to RA patients. Extending the needs assessment to encompass determinants of behavior gave us a good understanding of the causes of problems with health-related behaviors. The online program uses tailored behavioral change strategies too, which should improve the likelihood of RA patients’ ability to manage their disease. Another strength of intervention mapping is the use which is made of input from patients and health professionals. Integrating the experiences, knowledge, and visions of these diverse groups with scientific insights enabled us to develop a well-grounded intervention, tailored to the preferences and support needs of RA patients.

A second expected strength of the program is that the program is computer-tailored to the user’s level of self-efficacy. This ensures that RA patients receive material which is suited to their personal needs, and this may increase motivation to persist with exercises and strategies recommended therein. The online format has the further advantage that patients can use the program as often as they want or need. They can choose which modules to work through and can do so at their own speed, whenever they want.

A third expected strength of the program is the extent to which the online program can be integrated with regular nursing care. All the topics covered in the program fall within the scope of a specialist RA nurse’s expertise and can be discussed during appointments with a nurse. Specialist RA nurses can encourage RA patients to use the program and hopefully benefit by continuing to practice self-management.

The composition of the multidisciplinary panel might be considered a limitation of the study. All 5 RA patients had long disease duration and had found ways to cope with their illness and may not have been able to recall the problems they had in the early phase of the disease. However, in each meeting we asked the patients to try to remember how things had been when they were first diagnosed.

Another limitation might be the choice of the channel for communication relatively early in the development process. Instead of in step 4, we decided in step 1 to use an online program as communication channel. This influenced our choices at certain points in the development process, for example the choice for behavioral change strategies. This is in conflict with the concept of intervention mapping as an iterative process. However, choosing to use eHealth early on gave us the opportunity to learn about its pros and cons and how to deal with them during implementation.

### Conclusions

This article describes how to develop a self-management program in a structured way and could serve as a guide for the development of similar interventions. The study yielded an online, computer-tailored self-management program suitable for all RA patients. In the planned exploratory RCT, we will assess important outcomes and estimate the relevant effect sizes; this should be useful preparation for a larger RCT. The process evaluation will give us more insight into how RA patients use the program, which will inform future development of the program. We hope that this online self-management program will become one of the treatment options available to RA patients as part of an integrated disease management plan.

## Appendix

Multimedia Appendix 1 Example of a module.

## Abbreviations

RA: rheumatoid arthritis
RCT: randomized controlled trial
