# Supplementary material for: An Online Tailored Self-Management Program for Patients With Rheumatoid Arthritis: A Developmental Study
Source: JMIR Res Protoc. 2015 Dec 25;4(4):e140. doi: 10.2196/resprot.4571 (PMC4706640; doi:10.2196/resprot.4571)
Supplement: Multimedia Appendix 1 [file resprot_v4i4e140_app1.pdf]

Change objectives	Methods	Practical applications	Session
Patient knows the consequences of not setting his/her boundaries (knowledge)	Provide general information about health-related behavior	Texts providing information about the consequences of setting boundaries	1
	Increase memory and/or understanding of relevant information		
	Persuasive communication		
Patient knows why the consequences of not setting his/her boundaries arise (knowledge)	Provide general information about health-related behavior	Texts providing information about why setting boundaries is important	1
	Increase memory and/or understanding of transferred information		
	Persuasive communication		
Patient is conscious of the positive consequences of setting boundaries (attitude)	Provide general information about health-related behavior	Texts providing information about the positive consequences of setting boundaries (designed to persuade the user of the benefits of setting boundaries)	1
	Increase memory and/or understanding of relevant information		
	Persuasive communication		
Patient says that he/she is able to set boundaries (self-efficacy)	Provide instruction of how to perform behavior	Texts providing information on assertiveness and communicating verbally and nonverbally	2, 3
	Provide information about peer behavior	Quotes from other RA patients	2, 3, 4
		Videos with other RA patients	2
	Practice	Exercises to identify helpful or not-helpful thoughts	2
		Exercises focused on setting boundaries with 2 levels of difficulties	3, 4
	Use of social support	Exercises focused on practice with partner, family, or friends	3, 4
	Plan coping response	Using questions to let the patient identify barriers to setting boundaries and how to overcome these barriers	3, 4
	Reinforcement on behavioral progress	Praise for behavioral progress	3, 4
